# Genome-Wide Promoter Methylome of Small Renal Masses

**DOI:** 10.1371/journal.pone.0077309

**Published:** 2013-10-24

**Authors:** Ilsiya Ibragimova, Michael J. Slifker, Marie E. Maradeo, Gowrishankar Banumathy, Essel Dulaimi, Robert G. Uzzo, Paul Cairns

**Affiliations:** 1 Cancer Epigenetics Program and Kidney Keystone Program, Fox Chase Cancer Center, Philadelphia, Pennsylvania, United States of America; 2 Biostatistics and Bioinformatics, Fox Chase Cancer Center, Philadelphia, Pennsylvania, United States of America; 3 Department of Pathology and Kidney Keystone Program, Fox Chase Cancer Center, Philadelphia, Pennsylvania, United States of America; 4 Department of Surgery and Kidney Keystone Program, Fox Chase Cancer Center, Philadelphia, Pennsylvania, United States of America; Johns Hopkins University, United States of America

## Abstract

The majority of renal cell carcinoma (RCC) is now incidentally detected and presents as small renal masses (SRMs) defined as ≤4 cm in size. SRMs are heterogeneous comprising several histological types of RCC each with different biology and behavior, and benign tumors mainly oncocytoma. The varied prognosis of the different types of renal tumor has implications for management options. A key epigenetic alteration involved in the initiation and progression of cancer is aberrant methylation in the promoter region of a gene. The hypermethylation is associated with transcriptional repression and is an important mechanism of inactivation of tumor suppressor genes in neoplastic cells. We have determined the genome-wide promoter methylation profiles of 47 pT1a and 2 pT1b clear cell, papillary or chromophobe RCC, 25 benign renal oncocytoma ≤4 cm and 4 normal renal parenchyma specimens by Infinium HumanMethylation27 beadchip technology. We identify gene promoter hypermethylation signatures that distinguish clear cell and papillary from each other, from chromophobe and oncocytoma, and from normal renal cells. Pairwise comparisons revealed genes aberrantly hypermethylated in a tumor type but unmethylated in normal, and often unmethylated in the other renal tumor types. About 0.4% to 1.7% of genes comprised the promoter methylome in SRMs. The Infinium methylation score for representative genes was verified by gold standard technologies. The genes identified as differentially methylated implicate pathways involved in metabolism, tissue response to injury, epithelial to mesenchymal transition (EMT), signal transduction and G-protein coupled receptors (GPCRs), cancer, and stem cell regulation in the biology of RCC. Our findings contribute towards an improved understanding of the development of RCC, the different biology and behavior of histological types, and discovery of molecular subtypes. The differential methylation signatures may have utility in early detection and particularly differential diagnosis for prognostic stratification as well as identify novel gene and pathway targets for therapeutic intervention.

## Introduction

The incidence of renal cell carcinoma (RCC) continues to rise with approximately 58,000 new cases and 13,000 deaths projected in the US in 2013. Most organ-confined or locally advanced RCC are cured by surgical resection but patients with metastatic disease have a 5-year survival under 10% [1], [2]. The most common histological subtypes of RCC are clear cell (conventional) representing around 75–80% followed by papillary (10–15%) and chromophobe (5%). These subtypes have different genetics, biology and behavior [3]. Currently, the majority of RCC present as small renal masses (SRM) defined as ≤4 cm in size that are discovered incidentally. There is no obvious or defined precursor lesion to RCC other than RCC of smaller size [3]. About 15% of surgically resected renal masses are benign tumors and the frequency is higher in reported series of SRMs from Western countries [4]. Oncocytoma is the most common, comprising around 75%, of benign renal neoplasms [5], [6], [7]. In general, it is difficult to differentiate renal oncocytoma from RCC by existing radiological imaging techniques. Since active surveillance is an alternative to immediate surgical resection of RCC, markers that could differentiate between benign and malignant SRMs, as well as between the different RCC cell types, would be valuable for patient management.

Present knowledge of the molecular basis of cancer indicates that epigenetic alterations, e.g. aberrant promoter methylation, make an important contribution to the biological behavior of a tumor [8], [9]. Epigenetic inactivation by aberrant methylation of promoter CpG islands in the classical tumor suppressor genes *VHL* and *CDKN2A* or other genes such as *RASSF1A, GSTP1, MGMT* and *SFRP1* have been identified in RCC generally by a candidate approach [10]. Genome-wide methylation studies have mainly focused on clear cell RCC [11], [12] and SRMs have not yet been specifically examined. Further elucidation of the RCC methylome is important to identify new diagnostic, prognostic and predictive markers, to identify novel therapeutic targets, to assess potential targets for epigenetic drug therapy, to identify molecular subtypes, and to gain insight into the biology of RCC.

In this study, we used the Illumina Infinium HumanMethylation27 (HM27) beadchip containing 27578 probes with an average of 2 CpG sites derived from 14495 genes to examine and compare the genome-wide promoter methylation profiles of the main histological cell types of pT1a RCC, benign renal oncocytoma ≤4 cm in size and normal renal parenchyma cells.

## Materials and Methods

### Ethics Statement

The FCCC Institutional Review Board (IRB) approved the study and all patients provided written consent.

### Specimen Collection

Fifty primary RCC (25 clear cell, 15 papillary and 10 chromophobe) and 25 renal oncocytoma of ≤4 cm in size fresh-frozen tissue specimens banked from 2000–2009 were obtained from the Biospecimen Repository at Fox Chase Cancer Center (FCCC). The tumor specimens were reviewed with a pathologist for selection of tumor cell-enriched areas, i.e. >70% tumor cell nuclei, to be dissected out. Subsequent to the beadchip hybridization, 2 RCC were identified as 4.4 cm and 4.5 cm in size and are therefore pT1b [13]. These two specimens were retained in the study. One papillary tumor DNA failed a gender check with the HM27 X and Y chromosome probes and was removed as a specimen mismatch. Clinicopathological data for the tumors is given in Table S1. Four age-matched histologically normal kidney tissues from patients with no history of RCC or oncocytoma were obtained and reviewed by the pathologist to confirm the absence of neoplasia. The normal renal parenchyma specimens were obtained from 2 male and 2 female patients with a mean age of 66 years similar to the average age at diagnosis of RCC of 64 years from 2005–9 SEER data (http://seer.cancer.gov/statfacts/html/kidrp.html).

### DNA Extraction and Bisulfite Modification

DNA was extracted from fresh-frozen tissue using a standard technique of digestion with proteinase K followed by phenol-chloroform extraction and ethanol precipitation [14]. Genomic DNA (1 µg) from each sample was bisulfite modified using the EZ-DNA Methylation kit (Zymo Research Corporation, Irvine CA) according to the manufacturer’s protocol with the alternative incubation conditions as stated for use with the Infinium beadchip.

### Bead Chip Based DNA Methylation Analysis

Bisulfite treated DNA was isothermally amplified, enzymatically fragmented and hybridized to the BeadChip. We took care to distribute specimens of each histological type across different beadchips on different dates. We also ran 4 technical replicates on different beadchips on different dates. During hybridization, single-stranded DNA anneals to locus-specific DNA oligomers linked to individual bead types. Two bead types correspond to each CpG locus – one to the methylated and the other to the unmethylated state. Allele-specific primer annealing is followed by single-base extension using dinitrophenyl (DNP)- and biotin-labeled ddNTPs. After extension the BeadChip was fluorescently stained. The intensities of the beads’ fluorescence are detected by the Illumina BeadArray Reader and analyzed using Illumina’s BeadStudio software. The DNA methylation state, described as a β-value, varies between 0 (unmethylated) and 1 (fully methylated), representing the ratio of the intensity of the methylated bead type to the combined intensity of the methylated and unmethylated bead types at a CpG locus.

### Data Analysis

Methylation data were analyzed using the R/Bioconductor platform. The N-bead value averaged 18 bead replicates for each probe across all 78 beadchips. *ß*-values were used to exclude poor performance probes prior to comparison of the tumor groups. Up to 665 (of 27578) probes with missing *ß*-values (N-bead value = 0 in at least 1 beadchip) were removed. In addition, up to 7496 probes where *ß* = <0.1 in all 78 specimens were excluded. The exact number of probes removed depended upon the particular specimen groups compared. We also removed 1059 probes mapping to chromosomes X and Y as otherwise gender-specific methylation could skew the clustering analysis. We imposed cut-offs and ranked probes by the Wilcoxon-Rank sum test in a two group comparison with a *p* value <0.05 considered significant. Based on this approach the set of genes that are differentially methylated in RCC, oncocytoma and normal renal parencyma were ranked and thus prioritized for further analysis.

### Bisulfite Sequencing

A set of primers for the area containing the HM27 beadchip probe sequence, i.e. the identical CpG dinucleotides, was manually designed and a 200–400 bp size fragment was PCR amplified from bisulfite modified tumor DNA and histologically normal renal parenchyma DNA. The PCR product was loaded into a 1.5% agarose gel, then cut out and purified using the Qiagen gel purification kit (Qiagen, Valencia CA, USA). Direct sequencing was performed on an ABI 3100A capillary genetic analyzer and data analyzed by Sequencer Version 4.2.2 software. The primers used and the size of the amplicon for each gene analyzed are given in Table S2. Within the amplicon, cytosine bases outside CG dinucleotides served as a control for the efficiency of modification. A 50∶50 unmethylated:fully-methylated by M.SssI normal human genomic DNA control was used to identify PCR amplification or sequencing bias for each assay.

### Pyrosequencing

Primers for PCR amplification and pyrosequencing (Table S2) were designed using Biotage software (Qiagen, Valencia CA). For pyrosequencing analysis the Pyro Gold Reagent Kit (Qiagen, Valencia CA, USA) was used. An internal control, a C not in a CG dinucleotide, for the efficiency of modification was included in the assay for the *ATP2A3* gene promoter. A 50∶50 unmethylated:fully methylated DNA control was examined to identify amplification or sequencing bias for each assay.

### Quantitative Real-Time Methylation Specific PCR

Primer sequences to methylated DNA sequence were designed together with an internal Taqman probe labeled with FAM and MGB for each gene examined. *In vitro* methylated by M.SssI normal human genomic DNA, confirmed by bisulfite sequencing to show methylation for the gene to be analyzed, was used as a positive control. The concentration of this DNA was determined and a series of dilutions made for a standard curve. The unmethylated sequence of the *ACTINß* gene was used as a normalizing control. The percentage of methylated alleles was calculated for a gene based on the standard curve. An Applied Biosystems 7500 Real-Time PCR machine was used for qMSP and data analyzed with SDS 1.3.1 software. Primer and probe sequences for qMSP are given in Table S2.

### Pathway and Functional Theme Analysis

We used Ingenuity Pathway Analysis (Ingenuity Systems Redwood City CA USA) to identify significantly over-represented pathways, and DAVID [15], [16] for biological themes significantly over-represented, in the lists of differentially methylated genes between RCC, oncocytoma and normal renal parenchyma. IPA pathways or DAVID functional annotation clusters with enrichment scores ≥1.3 equivalent to a non-log scale *p* value <0.05 were considered significant [17].

## Results and Discussion

### Evidence for Distinct Gene Methylation Signatures Between Renal Cell Tumor Types

We first examined the Infinium HM27 beadchip data from the 78 renal specimens for consistency of assay performance. Specimens of each histological cell type were hybridized to different beadchips on different dates. Multi-dimensional scaling (MDS) analysis by each beadchip and date revealed no obvious batch effects (Figure S1a). Four technical replicates (5% of total specimens) were run on different beadchips and dates and the R^2^ correlation coefficient ranged from 0.985 to 0.993 (Figure S1b).

**Figure 1:**
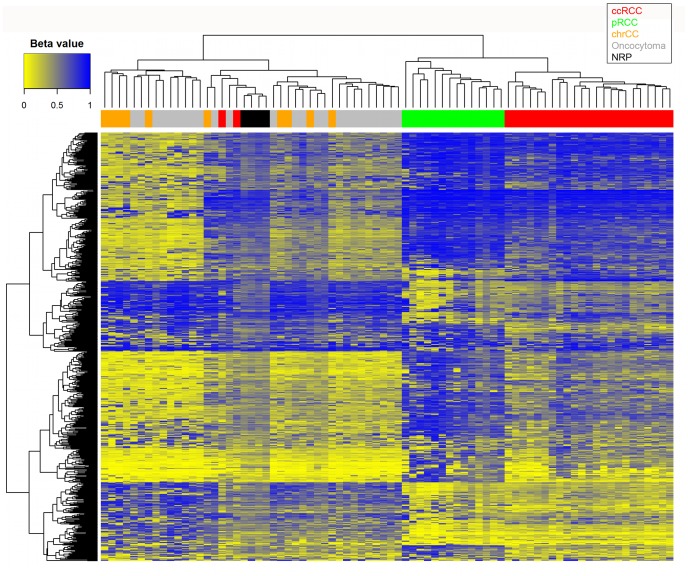
Unsupervised clustering analysis of the most differentially methylated probes. 74 renal tumors and 4 NRP identified by color top right. Top left is color scale unmethylated yellow (β = 0) - methylated blue (β = 1).

As a preliminary investigation we performed unsupervised two-dimensional hierarchical clustering of the most differentially methylated probes between all types of RCC, oncocytoma and the normal renal parenchyma (NRP) samples together. The most differentially methylated probes were selected by taking the 349 probes with maximum standard deviation (across all 78 samples), together with the top 349 probes by “normalized” standard deviation [18]. There were 231 probes in common in the two lists, giving a total of 467 probes in the combined list. Figure 1 shows that in regard to the histological cell types, 23 of 25 clear cell RCC (ccRCC) clustered together. We noted that the remaining 2 clear cell tumors were atypical: one was a cystic ccRCC and the other a ccRCC with sarcomatoid transformation. Within the cluster of 23 ccRCC there was no clear evidence for the 2 high stage or 5 high grade tumors to cluster together. All 14 papillary RCC (pRCC) clustered together. The 10 morphological type I and 4 type II [19] tumors did not cluster separately within the pRCC cluster. The 10 chromophobe RCC (chrRCC) and 25 oncocytoma were interspersed in two clusters. A subset of oncocytoma are multifocal and often of younger age [20]. The 25 oncocytoma in our study included 4 multifocal patients and 1 unifocal patient of a relatively young age (41 years) but these 5 oncocytomas were present in both clusters. Chromophobe RCC and oncocytoma are considered to have features in common. Both types are believed to originate from intercalated cells of collecting duct, the two types can be difficult to differentiate by histomorphology and immunohistochemical markers, and rare hybrid oncocytoma/chromophobe renal tumors have been described [21], [22]. The 4 NRP formed a tight cluster within one of the two chrRCC/oncocytoma clusters.

Overall, unsupervised clustering of the 467 most differentially methylated probes provided evidence for the two main subtypes of RCC, clear cell and papillary, having distinct methylation signatures to each other, and also to chromophobe RCC, oncocytoma, and normal renal parenchyma.

### Methylation Profiles May Identify Molecular Subtypes

Recent reports have further described [23] the CpG island methylator phenotype (CIMP) in colorectal cancer [24], [25] and identified CIMP in other types of cancer [26], [27]. Using a similar cut-off (SD>0.2) to these studies, we performed unsupervised clustering of the 54 most differentially methylated probes within the 25 ccRCC. Three main groups were evident on the heatmap (Figure S2), one with frequent methylation, one with infrequent methylation, and an intermediate group, similar to analysis of CIMP with HM27 in studies of colorectal, brain, and breast cancer [26], [27], [23]. The 2 high clinical stage tumors and 3 of 4 high grade tumors were in the frequent methylation group (cluster 1) while the group with least methylation (cluster 2) contained low grade, low stage ccRCC exclusively. This may provide preliminary evidence for association of CIMP with clinicopathological parameters or might reflect the accumulation of aberrant methylation with a more advanced grade or stage of malignancy as noted by TCGA [28]. Previous profiles of methylation in RCC have found some evidence for CIMP in RCC by clustering analysis [29], [11], [12]. The definition of CIMP in colorectal cancer was greatly aided by clear association with point mutation of *BRAF* [25]. The identification of CIMP in RCC will likely require more extensive study including association with genetic alterations, histopathology, tumor behavior and biology in order to produce a clearer definition of CIMP in this disease.

Further preliminary evidence that methylation signatures may identify subtypes was provided by one pRCC that clusters with, but stands out visually from, the other pRCC on the heatmap (far left of pRCC cluster in Figure 1). This tumor had been noted by the pathologist to have an atypical karyotype (35, −X, −1, −4, −6, −8, −9, −13, −14, −15, −18, −22) i.e. monosomy of several chromosomes more similar to chrRCC than to pRCC.

### Promoter CpG Island Hypermethylation between Renal Cell Tumors and Normal Renal Parenchyma

A more powerful approach to identification of differential methylation is to perform a two-group comparison with a test for statistical significance. We first examined probes with the most differential methylation between NRP cells from age-matched individuals with no history or evidence of RCC or oncocytoma and the three subtypes of RCC grouped together by the Wilcoxon Rank sum test in a two group comparison with *p*<0.05 considered significant. The Wilcoxon test was used because it does not make any distributional assumptions on the data. We imposed the additional condition that all 4 NRP DNA must have a *ß*-value under 0.15, i.e. to be considered unmethylated, for a probe to be included. We considered a probe hypermethylated if a tumor specimen *ß*-value showed a Δ>0.2 above the mean *ß*-value of the 4 NRP DNA. We chose these cut-offs for two reasons. First because Illumina reported that a Δ*ß* detection sensitivity of 0.2 could be detected with 95% confidence across more than 90% of probes in HM27 and also that the Δ*ß* sensitivity was higher (0.15) at the unmethylated state [30]. The second basis for our cut-offs may be best seen by example. The *VHL* tumor suppressor gene promoter is known to be aberrantly hypermethylated in 7–15% of sporadic clear cell RCC [31], [32], [33], [28]. If 1 of 2 alleles of *VHL* were hypermethylated and the other allele inactivated by point mutation (and unmethylated) in a primary ccRCC specimen with typical 30% normal cell contamination then the maximum percentage of methylated alleles would be 35%. A probe with a *ß*-value of 0.35 would be considered hypermethylated by our criteria of a *ß*-value ≥Δ0.2 above the mean of the NRP which cannot exceed 0.15.

**Figure 2:**
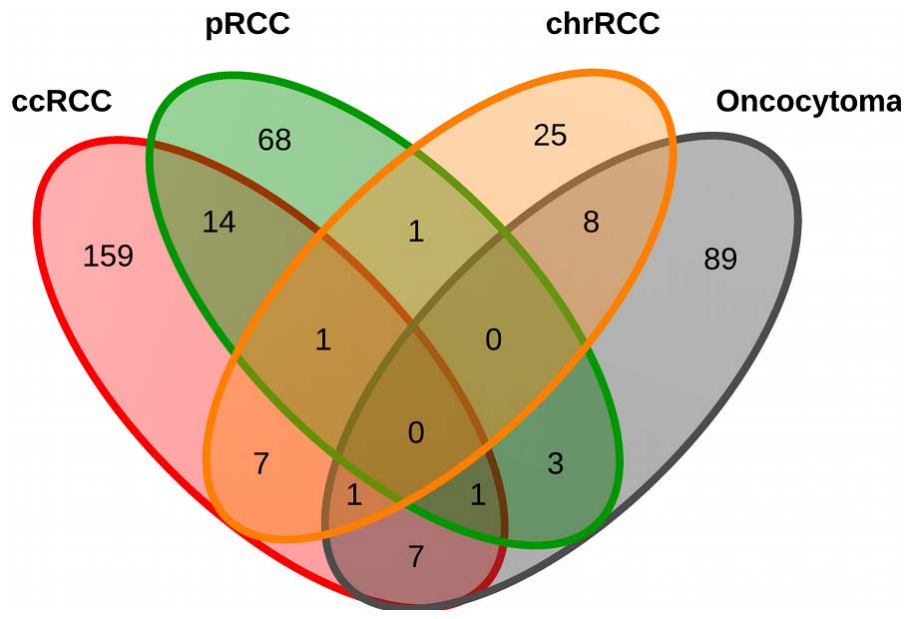
Venn diagram of the number of aberrantly hypermethylated genes in each renal tumor type compared to NRP.

The Wilcoxon test with these cut-offs generated a list of 335 probes hypermethylated in all RCC but unmethylated in NRP (*p*<0.05). Probes located in CpG islands were over-represented in the list at 94.6% (317) vs. 72.5% overall in the HM27 beadchip. Probes located in the proximal promoter region, defined here as 1 kb from the transcription start site (TSS), represented 95.2% (319) of the total. Taken together, 89.8% (301 probes from 292 genes) were located in a CpG island in the proximal promoter region. After this filtering, probes were ranked by highest frequency of hypermethylation within the tumor group. We then examined each tumor subtype separately against NRP (Table S3). For ccRCC 201 probes from 190 genes, for pRCC 89 probes from 88 genes, for chrRCC 44 probes from 43 genes, and for oncocytoma 112 probes from 109 genes fulfilled the above criteria. Approximately 1.7% (in ccRCC) to 0.4% (in chrRCC) of genes analyzed after filtering were hypermethylated. The proportion of genes hypermethylated in an individual tumor was much lower. In the 25 ccRCC, the number of genes hypermethylated per tumor ranged from 2–73 with a rounded mean of 27 and a median of 20 genes. The range, mean and median of the other tumor types are given in Table 1. The mean number of genes hypermethylated was higher in the more aggressive tumor types and lowest in benign oncocytoma. Importantly, for each tumor type the majority of aberrantly hypermethylated genes were unique to that type (Figure 2).

**Table 1:** PRC target genes are overrepresented in the renal cancer methylome.

	Total hyper-methylated genes	All 3 marks	Frequency (%)	*p*-value	1 or more marks	Frequency (%)	*p*-value
All RCC	292	24	8.2	0.0003	58	19.9	<0.0001
ccRCC	190	27	14.2	<0.0001	55	28.9	<0.0001
pRCC	88	13	14.8	<0.0001	25	28.4	<0.0001
chrRCC	43	7	16.3	0.0002	10	23.2	0.0051
Oncocytoma	109	11	10.1	0.0023	33	30.3	<0.0001

Proportion of PRC target genes among genes hypermethylated in renal cancer compared to ∼4% with 3 marks or 9.5% with 1 or more marks in human ES cells from Lee et al 2006 [48].

### Frequently Hypermethylated Genes in Renal Cell Tumors

**Figure 3:**
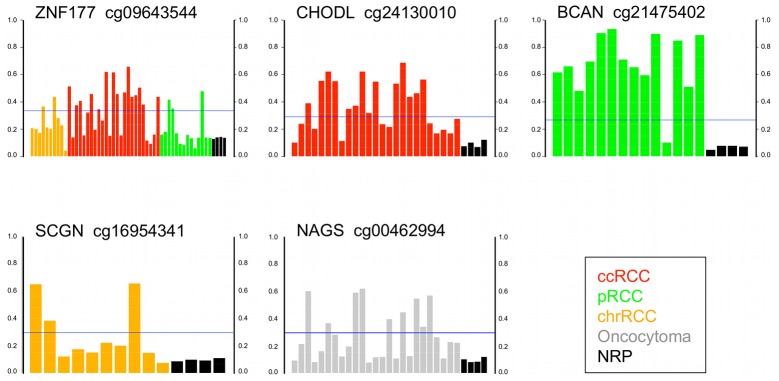
Barplots of genes aberrantly hypermethylated in RCC or oncocytoma. Y axis is Infinium β-value. Tumors above the horizontal blue line (delta β 0.2> mean of NRP) are hypermethylated for the gene compared to the unmethylated (β<0.15) 4 NRP. The gene name and Infinium probe ID are given above each barplot.

The majority of genes with a significant *p* value have not been previously reported as hypermethylated in RCC by candidate gene studies [34], [10]. Among the most frequently hypermethylated genes was *ZNF177*, which may be involved in regulation of transcription, hypermethylated in 20/49 (41%) RCC (Figure 3). The *GRIK1* gene was hypermethylated in 14/25 (56%) ccRCC. GRIK1 is of the ionotropic class of glutamate receptors, is expressed in normal kidney and maps to chromosome 21q22.11. Glutamate receptors are known to function in the mammalian brain and are activated in a variety of normal neurophysiologic processes. It is unclear what role GRIK1 may have in normal or neoplastic renal cells. However, another ionotrophic glutamate receptor *GRIN2A* was recently found to have frequent point mutation in melanoma [35], [36] though its function in this disease is unknown. Another frequently hypermethylated gene was *CHODL* the transmembrane protein chondrolectin hypermethylated in 14/25 (56%) ccRCC (Figure 3).

The *Brevican* (*BCAN)* gene was hypermethylated in 13 of 14 (93%) pRCC. Brevican is a proteoglycan that may be involved in growth and cell motility in the adult nervous system (Figure 3). The secretagogin protein (*SCGN*) was hypermethylated in 3/10 (30%) chrRCC. Secretagogin is a secreted calcium-binding protein located in the cytoplasm believed to be involved in KCL-stimulated calcium flux and cell proliferation. SCGN is reported to be expressed in ccRCC but not in non-ccRCC [37]. The *N-acetylglutamate synthase* (*NAGS*) gene was hypermethylated in 9/25 (36%) oncocytomas (Figure 3). *NAGS* encodes a mitochondrial enzyme that catalyzes the formation of N-acetylglutamate (NAG) from glutamate and acetyl coenzyme-A. NAG is a cofactor of carbamyl phosphate synthetase I (CPSI), the first enzyme of the urea cycle in mammals. NAGS may regulate ureagenesis which involves the metabolism of amino acids to urea.

Several genes with significant hypermethylation were previously reported to be aberrantly methylated in cancers other than RCC e.g. the *CALCA, CDH13, DCC, MYOD1, EDNRB, LAMC3, CDKN2B/p15, GATA4* and *CAV1* genes. Certain genes previously described as hypermethylated in RCC by candidate gene methodology were present at the frequency consistent with previous studies e.g. *MGMT, GSTP1, CDKN2A/p14* and *SFRP1* [31], [32], [38], [39]. In our study, although *VHL* was hypermethylated as expected in 2/25 (8%) ccRCC [31], [32], [33], [28] it was not statistically significant (*p* = 0.094). Two of the novel genes reported to have point mutation in ccRCC by TCGA [28] showed aberrant hypermethylation in our study. *SLC27A6* was hypermethylated in 8/25 ccRCC and *FBN2* was hypermethylated in 15/25 ccRCC although the probe was excluded from our final list because it is located more than 1 kb (1090 bp) distant to the TSS.

Two other studies have used Infinium HM27 technology to examine genome-wide methylation of RCC. The first study examined exclusively clear cell tumors and focused on defining a pre-cancerous methylation pattern in normal tissue adjacent to tumor and also a CIMP-positive group of ccRCC [11]. The other study identified 205 genes as hypermethylated in 38 RCC, described as clear cell or papillary without information on size, grade or stage. In a table of 60 genes from this study [12] the majority do not overlap with our list. While this paper was being written, Slater et al [40] reported an analysis of 20 chrRCC and 21 oncocytoma with Infinium 450 k technology. No information on tumor size or other histopathological information was provided. Disparities between our data and other studies are likely due to differences in the histopathology of tumor specimens, filtering of probes, methylation score cut-offs and statistical analysis.

### Pathways and Molecular Functions with Aberrant Methylation in Renal Cell Tumors

**Figure 4:**
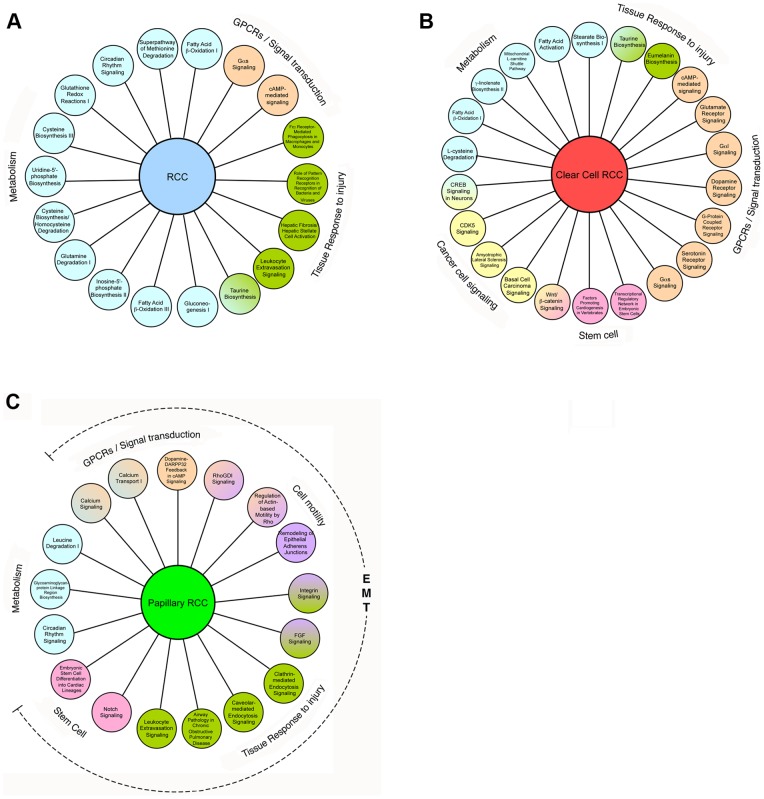
Pathways significantly overrepresented by aberrant gene methylation. IPA analysis of hypermethylated genes identified significant canonical pathways A) all RCC, B) ccRCC, C) pRCC.

An IPA analysis of genes hypermethylated in all RCC indicated 18 canonical pathways to be significantly over-represented (*p*<0.05). These pathways may be broadly categorized as 1) metabolism particularly of fatty acid, 2) tissue response to injury and 3) G-protein coupled receptors (GPCRs) and signal transduction (Figure 4A). In ccRCC, there were in addition, 4) signaling in proliferation, invasion and cancer, 5) embryonic stem cells and 6) renal hypertension (Figure 4B). RCC has been described as a disease of metabolism [41] and the similarity of a global gene expression profile of RCC to a wound injury/repair expression signature noted [42]. Papillary RCC (Figure 4C) was particularly enriched (13 of 17) for pathways implicated in epithelial to mesenchymal transition (EMT) [43].

Hypermethylated genes were also identified in signaling pathways of known relevance to the biology of RCC [44] including VEGF signaling, angiogenesis, and PTEN signaling (*FLT4, KDR*), Wnt/β-Catenin signaling (*AXIN1, CDKN2A, PPP2R1B, SOX11, WNT2*), PI3K/AKT signaling and mTOR signaling (*PPP2R1B*), IL-2 signaling (*SYK*), TGF-β signaling (*BMP4*), and NK-κβ signaling (*BMP4, FLT4, KDR, TIRAP*).

Gene Ontology (GO) biological processes highlighted by DAVID as enriched (*p*<0.05) in the analysis of genes hypermethylated in all RCC included regulation of transcription, cell motility and migration, negative regulation of metabolic and biosynthetic processes, and angiogenesis or blood vessel development.

It has been reported by several groups that stem cell polycomb group (PcG) target genes are over-represented in the set of genes that show aberrant hypermethylation in cancer [45], [46], [47]. Most studies have used the list of genes with the polycomb occupancy marks of the PRC2 subunits SUZ12 and EED associated with H3K27 methylation in human ES cells from Lee et al 2006 [48] where ∼4% (654 of 16,710) genes had all of the 3 marks examined (SUZ12, EED, H3K27) and 9.5% (1591 of 16,710) genes had 1 or more marks. We examined our lists of hypermethylated genes significant by Wilcoxon and found PcG genes to be significantly over-represented (Fishers exact test *p* <0.05) in each type of RCC as well as oncocytoma (Table 2). A recent study of the module of stem or progenitor cell genes with aberrant hypermethylation and downregulation in cancer noted the prominence of genes involved in neuronal development [49]. In our study of RCC, we also noted several canonical pathways or networks implicated in neuronal regulation. There are biological similarities between the migration of neurons and migration of neoplastic cells [50], [51].

**Table 2:** Number of genes hypermethylated in RCC and oncocytoma.

	Mean	% of Total	Median	Range
ccRCC	26.6	1.7	20	2–73
pRCC	21.6	0.8	15	4–30
chrRCC	5.2	0.4	4.5	1–12
Oncocytoma	5.4	1.03	4	0–17

De Carvalho et al examined the remaining DNA methylation in DKO cells compared to the parental HCT116 cells as an indicator of the minimum gene methylation, and therefore potential driver epigenetic alterations, necessary for tumor cell survival [52]. Several of the driver genes identified in that study, namely *CDO1, SLITRK1* and *CYYR1*, showed aberrant hypermethylation of promoter CpG islands in RCC in our study. De Carvalho et al noted that many driver genes hypermethylated in the colorectal tumor cells were GPCRs and, while this family of genes is large, it was also significantly overrepresented in our analysis of genes hypermethylated in RCC.

### Differential Methylation between RCC and Benign Oncocytoma

**Figure 5:**
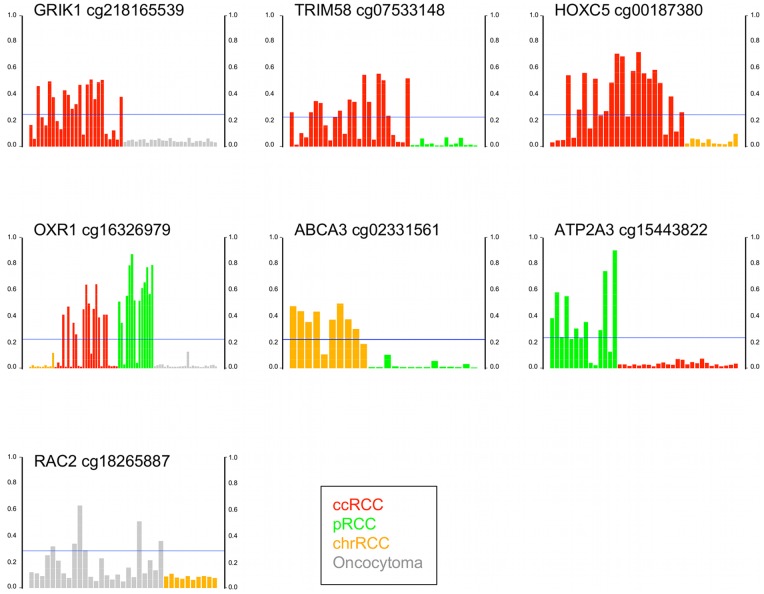
Panels of hypermethylated genes that discriminate between ccRCC and oncocytoma.

We examined a larger proportion of oncocytomas than represented in the population with SRMs because we were particularly interested in identification of a differential methylation signature between RCC and benign oncocytoma. We performed pairwise comparison between the tumor groups using the β<0.15 cut-off for genes unmethylated in every tumor of one group but hypermethylated in at least one tumor from the other group. The Wilcoxon test was significant for 538 probes from 497 genes hypermethylated in RCC and unmethylated in oncocytoma (Table S4). The *oxidation resistance protein 1* (*OXR1)* gene was hypermethylated in 24/39 (62%) ccRCC and pRCC but unmethylated in chrRCC or oncocytoma (Figure 5). OXR1 may be involved in resistance to damage by reactive oxygen species (ROS). In ccRCC, 485 probes from 437 genes were hypermethylated compared to oncocytoma (Table S5). One top-ranked gene was *GRIK1* hypermethylated in 14/25 (56%) ccRCC and unmethylated in oncocytoma (Figure 5). Other hypermethylated genes included several in the Wnt/β-catenin signaling pathway i.e. *SFRP1, SFRP5, DKK2* and *DKK3*. The *UCHL1* gene previously reported as methylated in RCC [53] was hypermethylated in 4/25 (16%) ccRCC and unmethylated in oncocytoma. Sixty genes were hypermethylated in oncocytoma but unmethylated in RCC (Table S6). Almost all of the 60 genes were hypermethylated in only 1 oncocytoma (of 25) indicating the considerable heterogeneity of gene hypermethylation in oncocytoma. For potential differential diagnosis, a panel of 6 genes would be positive for all 25 ccRCC and a separate panel of 8 genes positive for 24 of 25 oncocytomas (Figure 5).

**Figure 6:**
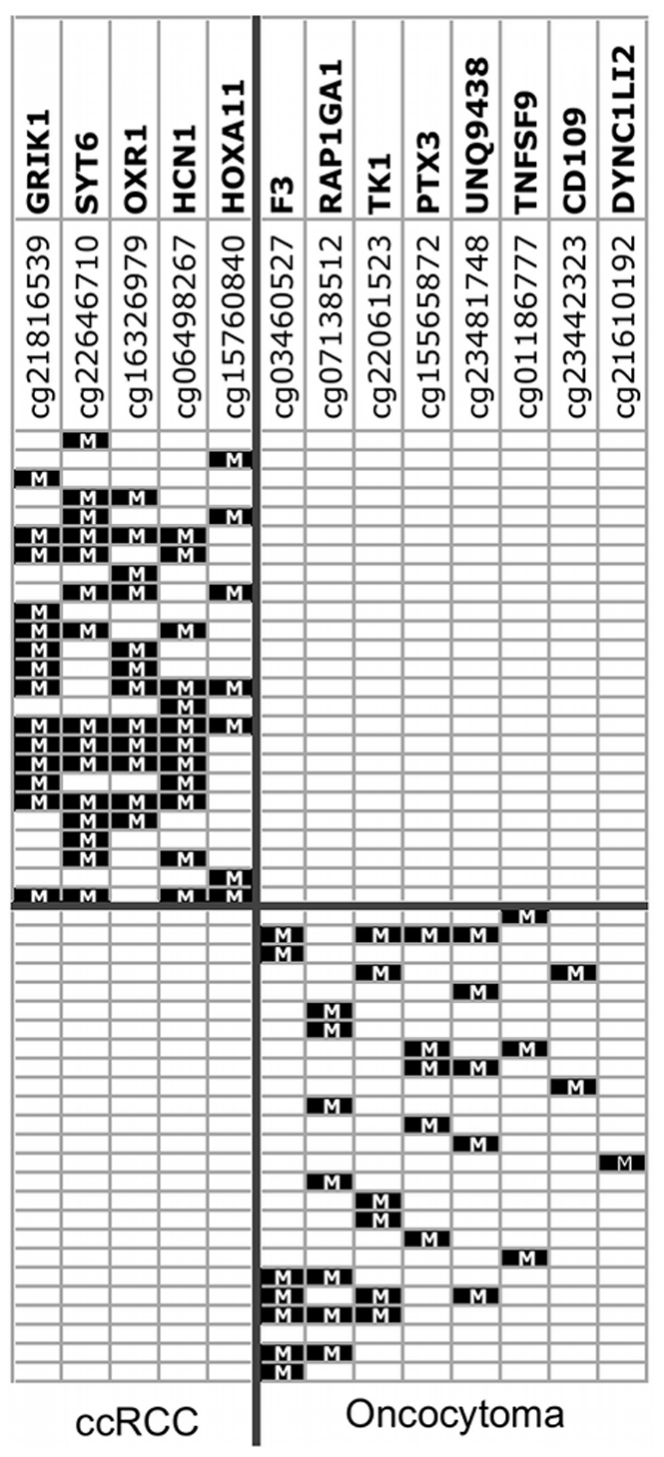
Barplots of genes differentially methylated between tumor types. Y axis is Infinium β-value. For each gene, tumors above the horizontal blue line (delta β 0.2> mean of comparison tumor type) are hypermethylated in the tumor type but unmethylated (β<0.15) in the comparison tumor type.

As described above, chrRCC and oncocytoma did not cluster separately by an unsupervised clustering analysis of the most differentially methylated probes across all 78 specimens. However, the two-group comparison by Wilcoxon identified 61 probes from 60 genes hypermethylated in chrRCC but unmethylated in 25 oncocytomas (Table S7). A further 80 genes were hypermethylated in oncocytoma and unmethylated in 10 chrRCC (Table S6) including *RAC2* hypermethylated in 6/25 (24%) of oncocytoma (Figure 6). *RAC2* is a small GTPase that belongs to the RHO sub-family. Small GTPases are important regulators of a wide variety of processes in the cell, including growth, differentiation, apoptosis, adhesion, movement and lipid vesicle transport [54]. Thus, the application of a rigorous condition that a probe be unmethylated in all of one tumor group combined with Wilcoxon identified a distinct methylation signature between chrRCC and oncocytoma. Verification of the distinct signatures in a larger and independent tumor series may identify the best candidate markers for differential diagnosis.

### Differential Methylation between Subtypes of RCC

The three main histological subtypes of RCC have different genetics, biology and behavior [3]. To examine differences in gene methylation between RCC types, we performed pairwise comparison between the tumor groups using the β<0.15 cut-off for genes unmethylated in every tumor of one group but hypermethylated in at least one tumor from the other group. For ccRCC vs. chrRCC 459 probes from 422 genes showed significance (Table S5). Top-ranked genes included the *FBLIM1* gene, located at chromosome 1p36.21, hypermethylated in 68% (17/25) ccRCC but unmethylated in 10 chrRCC. FBLIM1 links cell-extracellular matrix adhesions to the actin cytoskeleton and regulates cell morphology and motility. In esophageal cancer cells, over-expression of FBLIM1 inhibited cell invasion through degradation of β-catenin [55]. The *tripartite motif containing 58* (*TRIM58*) gene was hypermethylated in 13/25 (52%) ccRCC and unmethylated in pRCC (Figure 6). *TRIM58* is a protein coding gene of unknown function expressed in normal kidney. *ATP2A3* was hypermethylated in 9/14 (64%) pRCC and unmethylated in ccRCC (Table S8). The ATP2A3 enzyme catalyzes the hydrolysis of ATP coupled with the transport of calcium (Figure 6). The *OXR1* gene was hypermethylated in 12/14 (86%) of pRCC and unmethylated in 10 chrRCC. The *ABCA3* gene was hypermethylated in 8/10 (80%) chrRCC and unmethylated in all 14 pRCC (Figure 6) (Table S7). ABCA3 is a member of the ABC1 superfamily and involved in intracellular and extracellular transport of molecules including lipids such as cholesterol.

### Verification of Methylation Status by Independent Technologies

**Figure 7:**
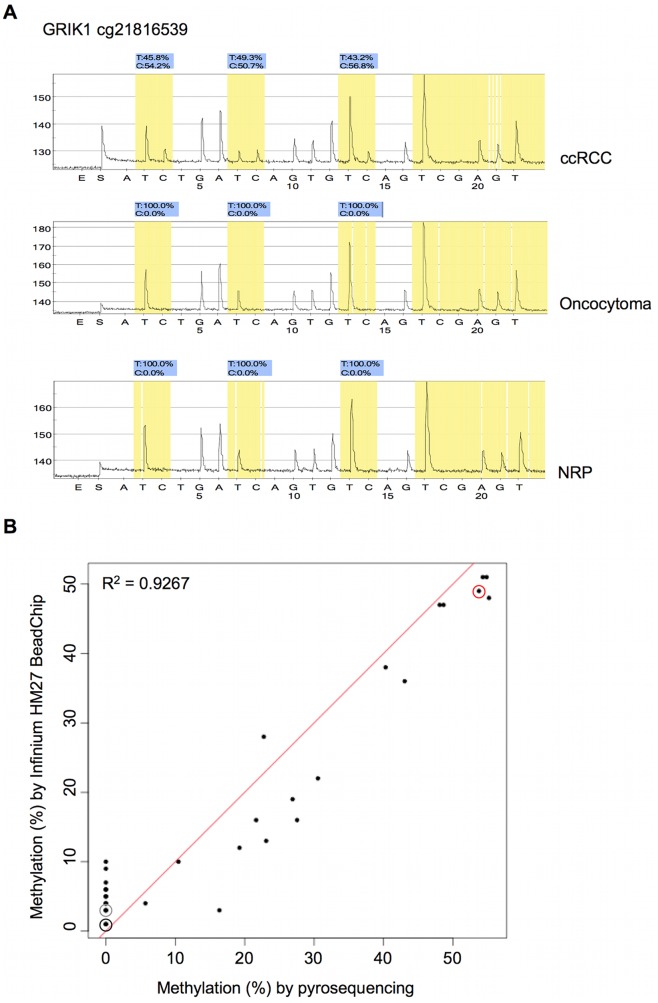
Verification of Infinium methylation score by pyrosequencing. A) Pyrograms of GRIK1 CpG loci hypermethylated in a ccRCC but unmethylated in an oncocytoma and NRP, B) Correlation between Infinium methylation score and pyrosequencing. The R^2^ is the Pearson coefficient. Circled red, gray, and black points correspond to ccRCC, oncocytoma, and NRP specimens respectively shown in A).

For verification of the accuracy of the Infinium HM27 *ß*-value as a methylation score, we selected several novel aberrantly methylated genes of interest for quantitation of methylation of the identical CG loci included in the Infinium probe by a gold standard independent technology i.e. pyrosequencing (*GRIK1, ZNF177, ATP2A3, OXR1*), direct bisulfite sequencing (*CHODL*), or quantitative methylation specific PCR (*BCAN*). We chose to examine gene probes located near to the TSS and within a bona fide CpG island [56] as well as with evidence of mRNA expression in normal renal cells by RNA sequencing data from the Illumina human body map project 2.0 [57]. Overall, we found excellent concordance between the Infinium *ß*-value and the methylation score of independent technologies (Figure 7) as reported in other studies [30], [18], [58].

### Hypermethylation of X-Chromosome Genes in Renal Cell Tumors

We analyzed X-chromosome probes separately from autosomal probes using Wilcoxon and the same criteria as before for males: the 2 normal renal males *ß*<0.15 and mean of male tumor group mean *ß*>0.15. For females, we modified the criteria to a probe was hypermethylated in a renal tumor from a female patient if the Δ*ß*>0.2 than the mean *ß*-value of the 2 female NRP specimens (Table S9). Two genes *KDM6A/UTX* and *KDM5C/JARID1C* recently identified with inactivating point mutation in RCC [59] are located on the X-chromosome and both are known to escape inactivation in normal cells of females [60], [61]. The probe for *KDM5C* is located inside a CpG island −49 bp of the TSS but showed no evidence of hypermethylation. The single probe for *KDM6A* is located outside (7770 bp upstream) of the promoter CpG island and was methylated (β = 0.69–0.78) in all 4 normal renal specimens. The most highly ranked probe within a bona fide CpG island and expressed in normal kidney [57], is from the *O-linked N-acetylglucosamine transferase* (*OGT*) gene which encodes the enzyme that catalyses O-GlcNAcylation important in cell signaling, transcriptional regulation and metabolism [62].

### Hypomethylation of Promoter CpG Islands in Renal Tumor Cells is Rare

We examined hypomethylation between NRP and all RCC by the Wilcoxon Rank sum test with inversion of the cut-offs used for hypermethylation: a probe showed *ß*>0.85 in all 4 NRP and hypomethylation was defined as Δβ≥0.2 below the mean *ß*-value of the 4 NRP. In all RCC, 48 probes were hypomethylated compared with NRP, 18 of which were located within a CpG island and 11 of these within 1 kb of the TSS (Table S10). We then examined hypomethylation in each tumor type separately. Overall, very few gene promoter CpG islands, 12 in ccRCC, 4 in pRCC, 2 in chrRCC and 3 in oncoytoma, showed hypomethylation by these criteria as expected from the current consensus that promoter CpG islands are generally unmethylated in normal cells [63]. One highly ranked gene was the *chemokine (C-X-C motif) receptor 5* (*CXCR5*) a membrane protein that may be involved in regulation in Burkitt’s lymphoma and B-cell differentiation. *CXCR5* was hypomethylated in 6/25 (24%) ccRCC and 6/14 (43%) pRCC.

### Hypermethylation of a Subset of miRNA Genomic Regions is Infrequent

The Infinium HM27 beadchip also includes probe sequences from the promoter regions of 100 of the known 900–1000 human miRNAs (http://www.mirbase.org/release 19). None appeared frequently methylated in RCC compared to NRP. The most hypermethylated miRNA was miR-564 hypermethylated (*ß* = 0.63) in 1 ccRCC and unmethylated in NRP (*ß* = 0.05–0.07). miR-564 has not been identified as downregulated in miRNA expression profiles of RCC [64], [65].

### Study Limitations

The HM27 beadchip includes one or more probes from 14,495 genes. The most recent NCBI CCDS database report has 18,606 genes [66], around 60% of which have a CpG island in the promoter region [30]. Thus, the HM27 has substantial but not complete coverage of human genes. The majority of HM27 probes are located in a promoter CpG island which, to date, is the area of the genome that has shown the clearest biological relevance when differentially methylated. However, on the HM27, there is no probe located in a bona fide CpG island of the promoter of certain genes known to be hypermethylated in RCC such as *CDKN2A/p16* or some genes of potential interest e.g. *PBRM1* [67]. Future analysis of the SRM methylome by Infinium 450 k beadchip [68] or whole genome bisulfite sequencing (WGBS) [69] will likely broaden coverage.

Two further points, other than the coverage of HM27, should be noted. The first point is that since we chose to examine SRMs, the majority of the RCC in our study are of low grade and low stage. Because cancer is generally believed to progress through the sequential accumulation of mutations [70], more advanced, i.e high grade and high stage RCC, might be expected to show higher frequencies of aberrant promoter methylation and/or may have additional genes methylated.

The second point is that the cell of origin of RCC or oncocytoma is not well studied. ccRCC and pRCC are thought to arise from cells in the proximal tubule while chrRCC and oncocytoma are believed to originate from intercalated cells in the distal nephron [71]. Since the progenitor cell of each histological type of RCC or oncocytoma is at present unclear, the relative frequency of the progenitor cell of each histological cell type in a piece of normal renal cortex is uncertain. In the unsupervised clustering heatmap (Figure 1) we noted that the 4 normals show a color pattern (percentage methylation) reflecting a mixed cell population. This is a confounding issue in studies of other types of epithelial cancer although rarely discussed [18]. The 4 age-matched normal specimens are useful to identify genes with renal tissue specific methylation or susceptible to age-related methylation in NRP cells. Comparison between the different renal tumor types should be more robust since tumors presumably originated from a single cell, although there can be more than one clonal population within a tumor, and a minimum level of 70% tumor cell content was imposed on tumor specimens for our study.

### Summary and Future Studies

We have examined the genome-wide promoter methylome of small renal masses and demonstrated reliable performance of the assay technology. Between 0.4–1.7% of genes were aberrantly hypermethylated in the renal tumor types, a proportion in line with the number of genes with exonic point mutation in ccRCC and other types of epithelial cancer [18], [72], [28]. Fewer genes still were commonly methylated (≥5% of tumors). Furthermore, it seems likely that a proportion of the gene hypermethylation will be passenger rather than driver events [73], [52] in the renal tumor cell. The majority of genes identified as hypermethylated are novel with several examples of involvement in pathways known to be important in RCC [44]. Novel pathways of metabolism, signal transduction and GPCRs, tissue response to injury, stem cell development and EMT are implicated by aberrant gene methylation. Functional validation of hypermethylated genes will first require evidence of association of hypermethylation with down-regulation of transcription in tumor cells and further analysis by standard growth and invasion assays will be necessary to provide support for a role in the biology of RCC. Several genes reported to be aberrantly hypermethylated in other cancer types but not RCC as well as a number of genes known to be hypermethylated in RCC were present in our analysis providing evidence for the specificity and potential of our survey. Distinct methylation signatures for each type of RCC as well as oncocytoma were revealed, and a considerable number of genes with differential methylation (hypermethylated in one tumor type but always unmethylated in another tumor type) identified including between chrRCC and oncocytoma. The majority of significantly hypermethylated genes in each tumor type were unique to that type. Preliminary evidence for different molecular subtypes within pT1 ccRCC was suggested by CIMP analysis.

The data here represent the promoter CpG island hypermethylome of pT1a RCC and oncocytoma of ≤4 cm in size (SRMs), the main type of renal tumor seen today in clinic. Because there is no defined precursor of RCC, aberrantly hypermethylated genes in small T1a tumors likely represent early events in the development of RCC. The differentially methylated genes identified, if supported by further study, may have clinical utility. Because RCC is generally only curable if detected before metastasis, we and others have previously demonstrated sensitive and specific detection of aberrant gene hypermethylation in urine and blood from patients with RCC as a strategy for early detection [74], [75]. In addition, patients diagnosed with SRMs are candidates for active surveillance where there would be benefit in the differential diagnosis of benign oncocytoma from RCC. There might be further advantage in the differential diagnosis of RCC cell type e.g. clear cell as more aggressive and also, in the future, of molecular subtypes within a particular RCC cell type i.e. more or less aggressive subsets of localized clear cell tumors. If further verification in additional specimen sets supports the specificity of a panel of methylated genes to one tumor type over another type, needle biopsy is an increasing option for patients with SRM diagnosed by imaging. Alternatively as a non-invasive approach, urine or blood specimens could be investigated for a cell type-specific methylation signature.

A subset of patients with metastatic RCC show no response to the current anti-VEGF or mTOR therapies while the patients that do respond inevitably develop resistance. The novel pathways with aberrant methylation highlighted in our study could identify new therapeutic targets. Lastly, RCC with methylated genes in pathways of known biological relevance or of particular molecular subtypes, e.g. CIMP-positive RCC, may indicate subsets of patients as candidates for epigenetic therapy.

## Supporting Information

Figure S1 **A) MDS analysis of Infinium HM27 data from different beadchips and dates.** The six batches are intermingled with the exception of a cluster on the right that represents the set of 14 pRCC that were run in batches A, B and E only. **B) Correlation plots of the 4 pairs of technical replicates.**
(TIF)

Figure S2 **CIMP analysis in ccRCC.** Unsupervised clustering of the most differentially methylated probes between the 25 ccRCC revealed 3 distinct clusters. Cluster 1 tumors have frequent hypermethylation i.e. potential CIMP. Cluster 2 has infrequent hypermethylation. Cluster 3 is intermediate. Top left is color scale unmethylated yellow (β = 0) - methylated blue (β = 1). The methylation status of the probe in NRP is shown separately at left. A horizontal red bar at far right indicates that the probe is located in a true CpG island.
(TIF)

Table S1 **Clinicopathological data for RCC and oncocytoma.** The Fuhrman nuclear grade and clinical stage of RCC are given. There were no stage II tumors in this study.
(DOC)

Table S2 **Primer and probe data.** Information on the CpG loci interrogated for each gene. Gene name, accession number and chromosomal location according to NCBI; Infinium probe ID; primer and probe sequences given 5′-3′, Y and R indicate degenerate T or C in forward and reverse primer respectively; amplicon size in base pairs.
(DOC)

Table S3 **Genes hypermethylated in renal tumors compared to NRP.** Column F Mean b-value_meth = mean of b-values from tumors with hypermethylation only. Column G Delta b-value = difference between mean b-value of tumors with hypermethylation only and mean b-value of 4 NRP.
(XLS)

Table S4 **Genes hypermethylated in RCC compared to oncocytoma.** Column F Mean b-value_meth = mean of b-values from tumors with hypermethylation only. Column G Delta b-value = difference between mean b-value of tumors with hypermethylation only and mean b-value of 25 oncocytomas.
(XLS)

Table S5 **Genes differentially hypermethylated in ccRCC.** Column F Mean b-value_meth = mean of b-values from ccRCC tumors with hypermethylation only. Column G Delta b-value = difference between mean b-value of ccRCC tumors with hypermethylation only and mean b-value of comparison tumor type.
(XLS)

Table S6 **Genes differentially hypermethylated in oncocytoma.** Column F Mean b-value_meth = mean of b-values from oncocytomas with hypermethylation only. Column G Delta b-value = difference between mean b-value of oncocytomas with hypermethylation only and mean b-value of comparison tumor type.
(XLS)

Table S7 **Genes differentially hypermethylated in chrRCC.** Column F Mean b-value_meth = mean of b-values from chrRCC tumors with hypermethylation only. Column G Delta b-value = difference between mean b-value of chrRCC tumors with hypermethylation only and mean b-value of comparison tumor type.
(XLS)

Table S8 **Genes differentially hypermethylated in pRCC.** Column F Mean b-value_meth = mean of b-values from pRCC tumors with hypermethylation only. Column G Delta b-value = difference between mean b-value of pRCC tumors with hypermethylation only and mean b-value of comparison tumor type.
(XLS)

Table S9 **X-chromosome genes hypermethylated in renal tumors.** Column F Mean b-value_meth = mean of b-values from tumors with hypermethylation only. Column G Delta b-value = difference between mean b-value of tumors with hypermethylation only and mean b-value of 4 NRP.
(XLS)

Table S10 **Genes hypomethylated in renal tumors compared to NRP.** Column F Mean b-value_meth = mean of b-values from tumors with hypomethylation only. Column G Delta b-value = difference between mean b-value of tumors with hypomethylation only and mean b-value of 4 NRP.
(XLS)
