# Supplementary figures and images for: Genome-Wide Promoter Methylome of Small Renal Masses
Source: PLoS One. 2013 Oct 24;8(10):e77309. doi: 10.1371/journal.pone.0077309 (PMC3811999; doi:10.1371/journal.pone.0077309)

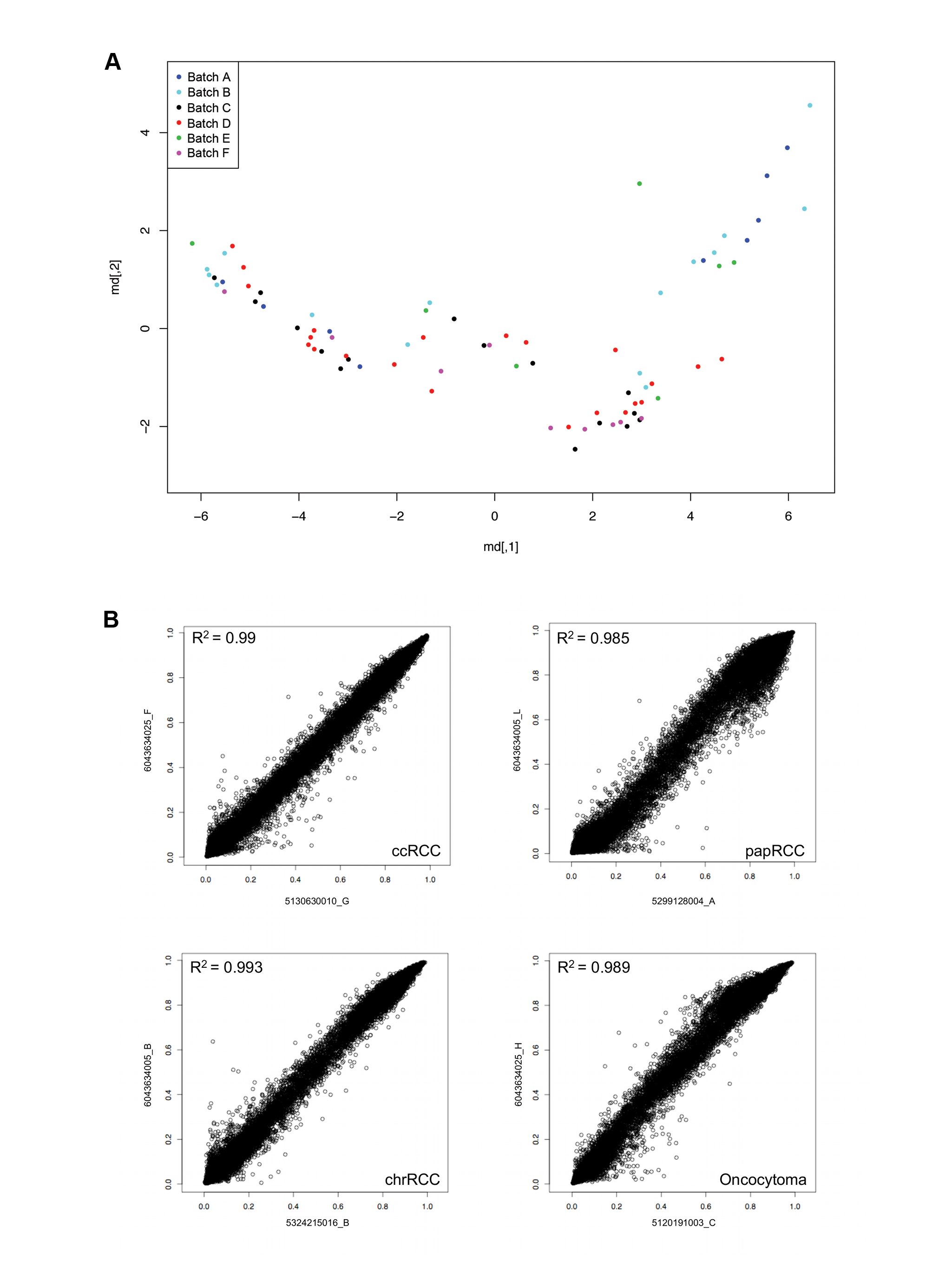

Supplement: Figure S1 — A) MDS analysis of Infinium HM27 data from different beadchips and dates. The six batches are intermingled with the exception of a cluster on the right that represents the set of 14 pRCC that were run in batches A, B and E only. B) Correlation plots of the 4 pairs of technical replicates. (TIF) [file pone.0077309.s001.tif]

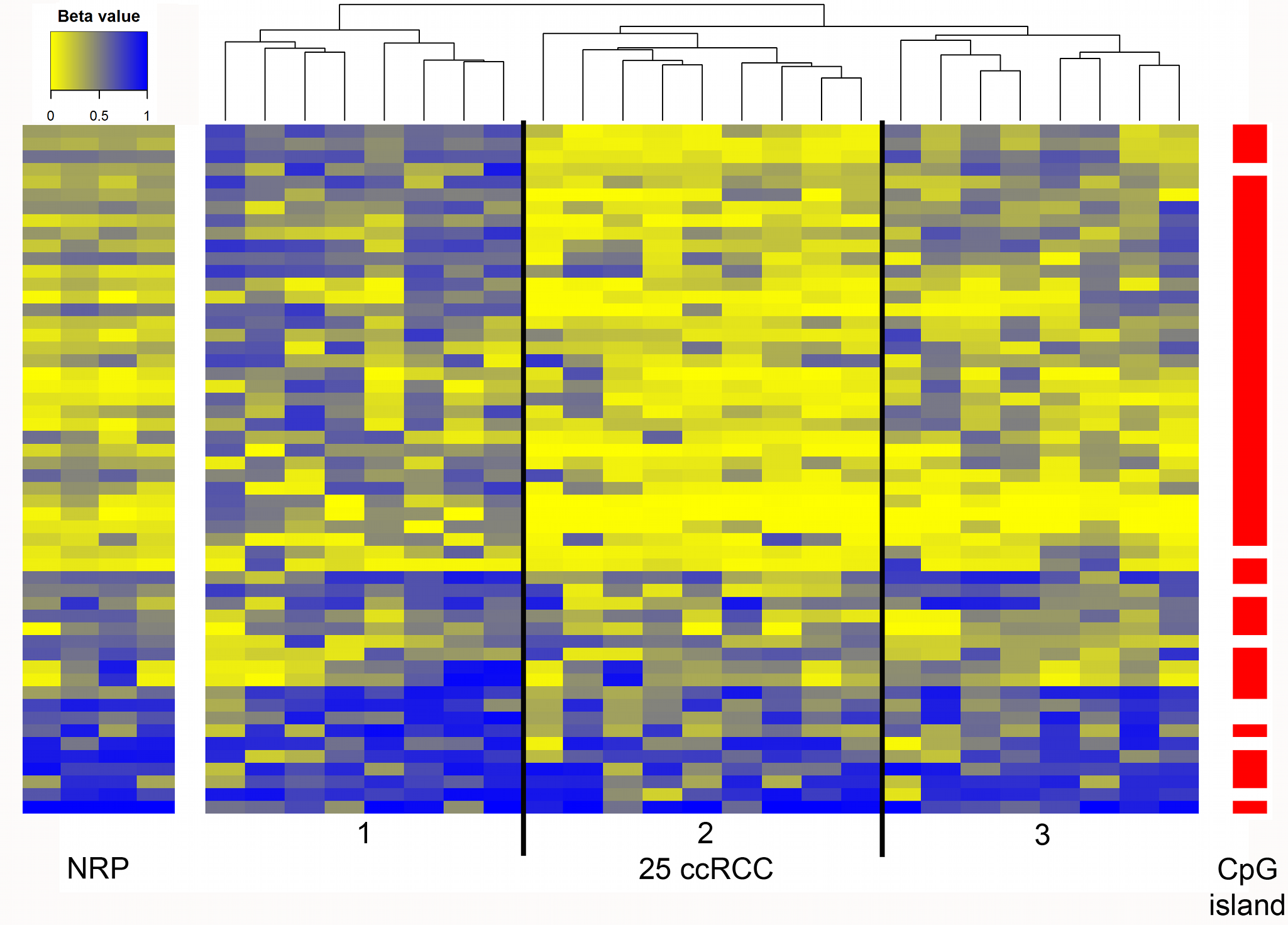

Supplement: Figure S2 — CIMP analysis in ccRCC. Unsupervised clustering of the most differentially methylated probes between the 25 ccRCC revealed 3 distinct clusters. Cluster 1 tumors have frequent hypermethylation i.e. potential CIMP. Cluster 2 has infrequent hypermethylation. Cluster 3 is intermediate. Top left is color scale unmethylated yellow (β = 0) - methylated blue (β = 1). The methylation status of the probe in NRP is shown separately at left. A horizontal red bar at far right indicates that the probe is located in a true CpG island. (TIF) [file pone.0077309.s002.tif]
