# Supplementary material for: Genome-Wide Promoter Methylome of Small Renal Masses
Source: PLoS One. 2013 Oct 24;8(10):e77309. doi: 10.1371/journal.pone.0077309 (PMC3811999; doi:10.1371/journal.pone.0077309)
Supplement: Table S1 — Clinicopathological data for RCC and oncocytoma. The Fuhrman nuclear grade and clinical stage of RCC are given. There were no stage II tumors in this study. (DOC) [file pone.0077309.s003.doc]

|  | Gender | Age Range | Median Age |
| --- | --- | --- | --- |
| ccRCC | M=16, F=9 | 33-85 | 56 |
| pRCC | M=8, F=6 | 38-84 | 58.5 |
| chrRCC | M=5, F=5 | 44-86 | 67.5 |
| Oncocytoma | M=16, F=9 | 41-85 | 70 |
|  |  |  |  |
|  | Stage I | Stage III | Stage IV |
| ccRCC |  |  |  |
| Grade I | 1 |  |  |
| Grade II | 18 | 1 |  |
| Grade III | 4 |  |  |
| Grade IV |  |  | 1 |
| pRCC |  |  |  |
| Low grade | 10 |  |  |
| High grade | 4 |  |  |
| chrRCC | 9 | 1 |  |
