# Supplementary material for: Genome-Wide Promoter Methylome of Small Renal Masses
Source: PLoS One. 2013 Oct 24;8(10):e77309. doi: 10.1371/journal.pone.0077309 (PMC3811999; doi:10.1371/journal.pone.0077309)
Supplement: Table S2 — Primer and probe data. Information on the CpG loci interrogated for each gene. Gene name, accession number and chromosomal location according to NCBI; Infinium probe ID; primer and probe sequences given 5′-3′, Y and R indicate degenerate T or C in forward and reverse primer respectively; amplicon size in base pairs. (DOC) [file pone.0077309.s004.doc]

| Gene name | Accession | Chromosomal location | Illumina probe ID | Primers / Probes | Amplicon size (bp) |
| --- | --- | --- | --- | --- | --- |
| GRIK1 | NM_000830.3 | 21q22.11 | cg21816539 | F - AGGGGAGGAGGAAAGTAAAGATT | 84 |
|  |  |  |  | R - AAACAAACCTAATAAACTCCAACAAATTA |  |
|  |  |  |  | PSQ - GAAAGTAAAGATTTAGAGAAG |  |
| ZNF177 | NM_003451.1 | 19p13.2 | cg09643544 | F - GAGTTGGGTAGTTTATTTTTTTTA | 109 |
|  |  |  |  | R - AAACAACAACCCTTTCTCA |  |
|  |  |  |  | PSQ - TGTAGTTGAGAAAGGGTTG |  |
| ATP2A3 | NM_005173.2 | 17p13.3 | cg15443822 | F – GGTTTTTTTTGAGGGTTTTAGAA | 76 |
|  |  |  |  | R - AAAAATAAAAACCCCAAAACTACTAAC |  |
|  |  |  |  | PSQ – TTTTTTGAGGGTTTTAGA |  |
| OXR1 | NM_181354.3 | 8q23 | cg16326979 | F - TTTGGGTTAGGTTTGATG | 82 |
|  |  |  |  | R - AAACTAAAACCCAAAAAATC |  |
|  |  |  |  | PSQ - TTGGGTTAGGTTTGATG |  |
| OXR1 | NM_181354.3 | 8q23 | cg17176732 | F -GGGTTAGGTTAGTTTTATTGG | 123 |
|  |  |  |  | R - ACATAAATATATTCTTCCTAAAACC |  |
|  |  |  |  | PSQ - AGTTTGTTTAAAAGTTATTA |  |
| BCAN | NM_198427.1 | 1q31 | cg21475402 | F - GTAGGTGAGTGTTTTCGTAGTTTCG | 123 |
|  |  |  |  | R - CCCTTCTCCCTATCCCCCA |  |
|  |  |  |  | FAM–TCGTTTGTTAGTTTTTGTTTCGA–MGBNFQ |  |
| CHODL | NM_024944.2 | 21q11.2 | cg24130010 | F -GAGGAGGYGGGTTGTTGATT | 278 |
|  |  |  |  | R - CAAAAACCCCRACTTAAACCAC |  |
